# A Patient with Polyuria and Polydipsia Following Silicone Injections

**DOI:** 10.34067/KID.0000000817

**Published:** 2025-10-30

**Authors:** Valeria De la Pena, Celine Garcia, Danny Haddad

**Affiliations:** 1 Rutgers Health, Newark, New Jersey; 2 Jersey City Medical Center, Jersey City, New Jersey

**Keywords:** activated vitamin D, calcium, CKD, clinical nephrology, complications, kidney stones, macrophages, mineral metabolism, nephropathy, obstructive nephropathy

## Abstract

**Figure:**
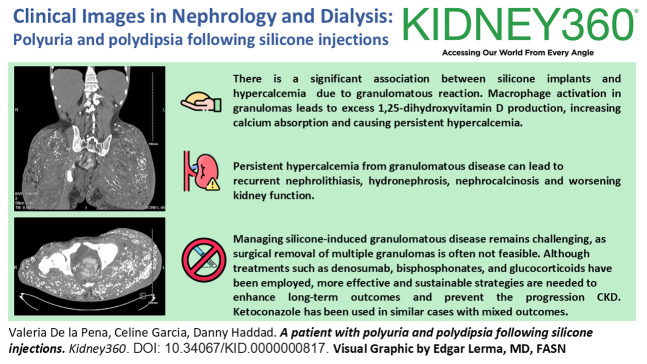

## Case Description

**Figure 1:**
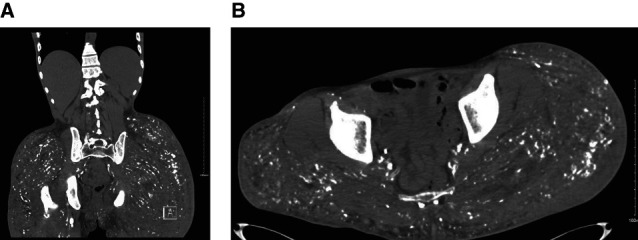
**Calcified granulomas on silicone injections.** CT scan of the abdomen and pelvis shows scattered calcified granulomas throughout the bilateral gluteal subcutaneous fat and muscles on coronal (A) and axial (B) images. CT, computed tomography.

A 52-year-old transgender woman with a medical history of asthma, hypertension, and silicone injections in the glutes presented with polyuria, polydipsia, and constipation. Bloodwork showed serum calcium of 16 mg/dl, BUN 38 mg/dl, and serum creatinine 2.03 mg/dl. Further workup revealed low parathyroid hormone 6.3 pg/ml, low parathyroid hormone-related-peptide 16 pg/ml, low 25-hydroxyvitamin D 15 ng/ml, and elevated 1,25-dihydroxyvitamin D 66 pg/ml. The patient was treated with intravenous fluids, zoledronic acid, and calcitonin for 5 days and discharged on alendronate 70 mg weekly. Four months later, the patient was hospitalized with symptomatic hypercalcemia. Further workup revealed negative serum protein electrophoresis and immunofixation, elevated lactate dehydrogenase 234 U/L, and elevated angiotensin converting enzyme levels 162.9 U/L, suggesting granulomatous disease. A computed tomography scan of the abdomen/pelvis showed infiltration throughout the bilateral gluteal subcutaneous fat with scattered calcified granulomas (Figure 1, A and B). A bone scan showed diffuse radionuclide accumulation in the glutes, corresponding with soft tissue attenuation and scattered calcifications. She was then started on denosumab 120 mg monthly with modest improvement in serum calcium levels. She also underwent a 4-month trial of prednisone 10 mg daily, during which no significant decrease in calcium was noted and kidney function persistently declined, which is the reason why it was discontinued. The patient continued on denosumab for 2 years; unfortunately, she continued having episodes of intermittent severe hypercalcemia, leading to recurrent nephrolithiasis and hydronephrosis, which further compromised the kidney function. Serum calcium levels began to stabilize by the third year of treatment; unfortunately, this coincided with the patient progressing to CKD stage 4.

## Discussion

This case underscores the importance of considering rare causes of recurrent hypercalcemia, such as tissue reactions with multiple granuloma formation associated with silicone injections. Granuloma formation due to the deposited silicone is associated with hypercalcemia, which can lead to prolonged hypercalciuria and CKD.^1^ An immune reaction to silicone leads to hypercalcemia by increasing calcium absorption through the production of 1,25 vitamin D3.^2^ In addition to the therapies provided in this case, normalization of serum calcium could be related to the development of advanced CKD. Owing to the challenge of removing extensive granulomas, the development of alternative management strategies to lower serum calcium is crucial.

## Teaching Points

- There is a significant association between silicone implants and hypercalcemia due to granulomatous reaction. Macrophage activation in granulomas leads to excess 1,25-dihydroxyvitamin D production, increasing calcium absorption and causing persistent hypercalcemia.
- Persistent hypercalcemia from granulomatous disease can lead to recurrent nephrolithiasis, hydronephrosis, and worsening kidney function, as observed in this patient who progressed to CKD stage 4 despite treatment.
- Managing silicone-induced granulomatous disease remains challenging, as surgical removal of multiple granulomas is often not feasible. Although treatments such as denosumab, bisphosphonates, and glucocorticoids have been used, more effective and sustainable strategies are needed to enhance long-term outcomes and prevent the progression CKD. Ketoconazole has been used in similar cases with mixed outcomes, although it was not used in this patient's treatment.

## Supplementary Material

## References

[1] Kozeny GA Barbato AL Bansal VK Vertuno LL Hano JE. Hypercalcemia associated with silicone-induced granulomas. N Engl J Med. 1984;311(17):1103–1105. doi:10.1056/NEJM198410253111707 6548295

[2] Dangol GMS Negrete H. Silicone-induced granulomatous reaction causing severe hypercalcemia: case report and literature review. Case Rep Nephrol. 2019;2019:9126172. doi:10.1155/2019/9126172 30729052 PMC6341244

