# Supplementary material for: A Patient with Polyuria and Polydipsia Following Silicone Injections
Source: Kidney360. 2025 Oct 30;6(10):1826–7. doi: 10.34067/KID.0000000817 (PMC12778019; doi:10.34067/KID.0000000817)
Supplement: Supplementary file 1 [file kidney360-6-1826-s001.pdf]

ASN
American Society of Nephrology
JASN
Journal of the American Society of Nephrology
KIDNEY360
Accessing Our World From Every Angle
CJASN
Clinical Journal of the American Society of Nephrology

ASN Journal Disclosure Form

As per ASN journal policy, I have disclosed any financial relationships or commitments I have held in the past 36 months as included below. I have listed my Current Employer below to indicate there is a relationship requiring disclosure. If no relationship exists, my Current Employer is not listed.

A. De la Pena has nothing to disclose.

I understand that the information above will be published within the journal article, if accepted, and that failure to comply and/or to accurately and completely report the potential financial conflicts of interest could lead to the following: 1) Prior to publication, article rejection, or 2) Post-publication, sanctions ranging from, but not limited to, issuing a correction, reporting the inaccurate information to the authors' institution, banning authors from submitting work to ASN journals for varying lengths of time, and/or retraction of the published work.

Name: Anna Valeria De la Pena
Manuscript ID: K360-2025-000172R1
Manuscript Title: A patient with polyuria and polydipsia following silicone injections
Date of Completion: April 8, 2025
Disclosure Updated Date: May 19, 2024

ASN American Society of Nephrology
JASN Journal of the American Society of Nephrology
KIDNEY360 Accessing Our World From Every Angle
CJASN Clinical Journal of the American Society of Nephrology

ASN Journal Disclosure Form

As per ASN journal policy, I have disclosed any financial relationships or commitments I have held in the past 36 months as included below. I have listed my Current Employer below to indicate there is a relationship requiring disclosure. If no relationship exists, my Current Employer is not listed.

C. Garcia reports the following:
Employer: Jersey City Medical Center

I understand that the information above will be published within the journal article, if accepted, and that failure to comply and/or to accurately and completely report the potential financial conflicts of interest could lead to the following: 1) Prior to publication, article rejection, or 2) Post-publication, sanctions ranging from, but not limited to, issuing a correction, reporting the inaccurate information to the authors' institution, banning authors from submitting work to ASN journals for varying lengths of time, and/or retraction of the published work.

Name: Celine Garcia
Manuscript ID: K360-2025-000172R1
Manuscript Title: Celinegarciasmc123@gmail.com
Date of Completion: April 7, 2025
Disclosure Updated Date: April 7, 2025

ASN American Society of Nephrology
JASN Journal of the American Society of Nephrology
KIDNEY360 Accessing Our World From Every Angle
CJASN Clinical Journal of the American Society of Nephrology

ASN Journal Disclosure Form

As per ASN journal policy, I have disclosed any financial relationships or commitments I have held in the past 36 months as included below. I have listed my Current Employer below to indicate there is a relationship requiring disclosure. If no relationship exists, my Current Employer is not listed.

D. Haddad reports the following:
Employer: Fresenius Kidney Care; Consultancy: Astrazeneca; Ownership Interest: Aurinia, Amarin, Novavax, Astrazeneca; Honoraria: Astrazeneca; Advisory or Leadership Role: national kidney foundation, fresenius kidney care; and Speakers Bureau: Astrazeneca.

I understand that the information above will be published within the journal article, if accepted, and that failure to comply and/or to accurately and completely report the potential financial conflicts of interest could lead to the following: 1) Prior to publication, article rejection, or 2) Post-publication, sanctions ranging from, but not limited to, issuing a correction, reporting the inaccurate information to the authors' institution, banning authors from submitting work to ASN journals for varying lengths of time, and/or retraction of the published work.

Name: Danny Haddad
Manuscript ID: K360-2025-000172R1
Manuscript Title: A patient with polyuria and polydipsia following silicone injections
Date of Completion: April 8, 2025
Disclosure Updated Date: April 8, 2025
